# Preemption in State Tobacco Minimum Legal Sales Age Laws in the US, 2022: A Policy Analysis of State Statutes and Case Laws

**DOI:** 10.3390/ijerph20116016

**Published:** 2023-05-31

**Authors:** Page D. Dobbs, Ginny Chadwick, Eric Crosbie, Jessica Breslin, Lisa Henriksen

**Affiliations:** 1 Department of Health, Human Performance and Recreation, University of Arkansas, 308A HPER Building, Fayetteville, AR 72701, USA; 2 Family and Community Medicine, University of Missouri, Columbia, MO 65211, USA; ginnychadwick@brandeis.edu; 3 School of Public Health, University of Nevada Reno, Reno, NV 89557, USA; ecrosbie@unr.edu; 4 ChangeLab Solutions, Oakland, CA 94612, USA; jbreslin@changelabsolutions.org; 5 Stanford Prevention Research Center, Stanford University School of Medicine, Palo Alto, CA 94305, USA

**Keywords:** preemption, tobacco control, tobacco law, tobacco policies, tobacco minimum legal sales age, Tobacco 21

## Abstract

Preemptive statutory language within tobacco minimum legal sales age (MLSA) laws has prohibited localities from enacting stricter laws than state statutes. With the recent uptake of state Tobacco 21 laws in the US, the current landscape of preempted MLSA laws is unknown. This study sought to update the status of preemption in MLSA laws enacted in US states between 2015–2022. A public health attorney reviewed state tobacco MLSA laws (*n* = 50) and state tobacco control codes, searching for language regarding preemption. When statutes were unclear, case law was reviewed by examining local ordinances that were invalidated by state court decisions. Overall, 40 states enacted Tobacco 21 laws, seven of which expanded or introduced preemption when they increased the MLSA; a total of 26 states (52%) included preemption. Six states (12%) retained ‘savings clauses’ included in the MLSA prior to Tobacco 21, and 18 states (36%) did not mention preemption. Based on the precedent set by state courts, eight of these 18 states may preempt localities from raising their MLSA. Historically, preemption has slowed the diffusion of best practices in tobacco control, and once implemented, the laws are difficult to repeal. The recent expansion of preemption could inhibit the evolution, development, and implementation of effective tobacco control policies.

## 1. Introduction

On 20 December 2019, the U.S. Congress raised the minimum legal sales age (MLSA) for tobacco products from 18 to 21 years of age. Although many believed the federal law applied to all states automatically and would decrease tobacco initiation and use among youth [1], enforcement practices varied by state, and states were not required to raise their own MLSA. When the 1992 Synar Agreement was passed, states were required to show they were in compliance with a MLSA law of 18 within five years or risk losing a portion of their Substance Abuse and Mental Health Services (SAMHSA) funding [2]. Similarly, the federal Tobacco 21 law did not mandate states to raise their MLSA but required compliance with federal law. Thus, 21 states passed their own Tobacco 21 laws following the federal MLSA increase, many of which provided instructions for implementation and enforcement (e.g., raised the age for underage decoys used for compliance checks of tobacco retailers). As of October 1 2022, all but ten states had passed such laws [3].

A key barrier to enacting and enforcing Tobacco 21 laws at the local level, which often includes strong policy components, is preemption [4,5]. Floor preemption is a foundational policy that provides a minimum standard (e.g., MLSA that allows local policies to raise but not lower the sales age). Ceiling preemption, referred to as ‘preemption’ throughout this paper, initially emerged as a public health threat to local tobacco control and firearms laws during the late 1980s. [6] It occurs when higher levels of government prohibit stricter policies from being passed by lower levels of government (e.g., federal to state; state to city) [6,7], and it has been a discussion of prevention via punitive preemption in other countries (i.e., Australia) [8]. Although floor preemption can be used to protect public health, preemption has recently become a barrier to introducing innovative local initiatives, such as taxes on sugary drinks, banning plastic bags, COVID restrictions, establishing minimum wage standards, and protecting LGBTQ rights [9,10]. Once in place, preemption is difficult to remove and inhibits the diffusion of best practices.

While preemption has recently become a threat to other areas of public health, it remains a key strategy by the tobacco industry to prohibit or stall evidence-based efforts (e.g., increase excise taxes, decrease youth access, adopt comprehensive smoke-free air laws) that decrease tobacco use and exposure [11]. Additionally, some states (e.g., Vermont) enforce what is known as “Dillon’s Rule”, where local government authorities are limited to only what is expressly granted to them in state law [12]. Thus, even though these state laws do not expressly proclaim there is preemption, the precedent set by state courts limits localities from increasing their MLSA or strengthening enforcement strategies included in the law. Alternatively, states can include a ‘savings clause’ that explicitly provides authority to lower-level governments to pass more stringent MLSA laws than state or federal laws [4,13], a provision that protects against legal challenges that are determined by state-level courts.

In 2014, 19 states preempted local jurisdictions from enacting stricter tobacco MLSA restrictions than the state, and eight states clearly granted local authority by expressing that the state law did not preempt localities from passing MLSA laws [4]. While this baseline study provided an essential understanding of the landscape of state MLSA preemption, the subsequent enactment of state Tobacco 21 laws requires updated findings. Furthermore, within examining state case law, it is unclear how state MLSA laws are being interpreted by state-level courts, which could alter the reality of preemption in practice from what has been reported in state statutes. Thus, the current study aims to update a comprehensive assessment of state-level preemption of MLSA laws by examining (1) the inclusion/exclusion of preemption language in state MLSA laws and (2) the validation/invalidation of local ordinances to determine if state courts have determined local authorities lacked the authority to enact MLSA or substance-related age restrictive laws that are stricter than the state.

## 2. Materials and Methods

**Figure 1:**
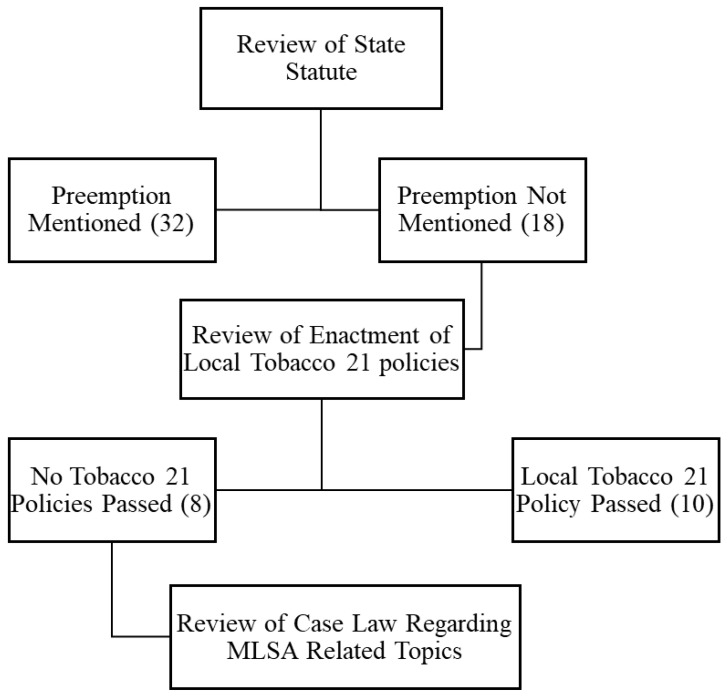
Review Process of Preemption Expressed in State MLSA Statutes and Local Case Laws.

State Tobacco 21 laws passed by 1 October 2022 (*n* = 40) were identified via state legislative websites using a procedure used in previous research [14]. Using a systematic process, a public health attorney reviewed the MLSA of all states looking for expressed age restriction preemption language, see Figure 1. This included reviewing state statutes or bills with the term ‘tobacco’ and then reviewing for minimum legal sales age and/or preemption of local ordinances. Using the language included in state laws, states were categorized into three groups: preemption expressed, expressly not preempted, and preemption not mentioned. State laws categorized as ‘preemption not mentioned’ were reviewed using the American Lung Association’s State Legislated Actions on Tobacco Issues [SLATI], and we searched for the enactment of unchallenged local Tobacco 21 laws, which would have demonstrated a lack of preemption [5]. Next, we reviewed case laws within each of the remaining states using Westlaw to identify references for court decisions related to implementation. Case laws were reviewed for ordinances that had been validated/invalidated by state courts due to the interpretation of preemption by state laws and challenges to implementation in states where courts interpreted implied preemption. These searches were narrowed using the term ‘tobacco’. When no tobacco cases existed, local ordinances that applied to alcohol were reviewed to determine the precedent of court decisions about age-restricted substances or regulations that were designed to protect public health.

## 3. Results

### 3.1. Inclusion/Exclusion of Preemption

By 1 October 2022, 40 states had passed Tobacco 21 laws; 19 before and 21 after the federal law was enacted on 20 December 2019. Although no states removed preemption of the MLSA with the adoption of Tobacco 21, seven states (Arkansas, Florida, Hawaii, Idaho, Massachusetts, Texas, and Utah) introduced or expanded preemption. As seen in Table 1, this raised the number of states that included explicit preemption to 26 at the time data were collected; 21 passed a Tobacco 21 law. Eighteen states did not mention preemption as it related to MLSA and local governments, 14 of which had enacted Tobacco 21.

### 3.2. Defined Tobacco 21 MLSA Law

In 2020, Mississippi passed a law that raised the minimum legal purchase age for all tobacco products to 21 and the MLSA of alternative nicotine products (not combustible cigarettes) to 21 years of age [2]. All other states either included novel tobacco and nicotine products (including e-cigarettes) in their tobacco product definition or explicitly stated that electronic nicotine delivery systems were included in the MLSA law. Thus, Mississippi was not included as a Tobacco 21 state in our analysis.

### 3.3. Savings Clause

Between 2016 and 2022, six states (Colorado, Maine, Minnesota, Missouri, New Hampshire, and North Dakota) retained a ‘savings clause’ in their existing statutes that gave authority to lower jurisdictions to pass stricter MLSA laws than the state. Five of these states (all except Missouri) passed Tobacco 21 laws; however, no state introduced a ‘savings clause’ with the adoption of their statewide Tobacco 21 law. Texas was the only state that both removed an existing ‘savings clause’ and introduced preemption of local authority to enact stricter MLSA laws than the state in their Tobacco 21 bill that was subsequently signed into law.

### 3.4. Purchase to Sales Transition

While MLSA laws hold retailers accountable for enforcing tobacco control measures, purchase laws redirect the responsibility from tobacco retailers to underage purchasers. The state of Colorado adopted language that transferred restrictions from underage individuals who attempted to possess or purchase tobacco products to the retailer. To do this, policymakers changed the language within the state law from “purchase and possession” to “sale of” and removed penalties for underage purchasers. The revisions also retained the ability for localities to adopt more stringent laws than the state. In addition, Colorado prohibited localities from passing purchase policies that set the age below 21 years. Table 2 provides an example of the language that transferred Colorado’s purchase law to a sales law and an example of the language that prohibited purchase laws for those under 21 years of age.

### 3.5. Case Law Analysis

Among states without clear preemption language (*n* = 18), ten states (Alaska, Arizona, Connecticut, Illinois, Kansas, New Jersey, New York, Ohio, Oregon, and Rhode Island) enacted the Tobacco 21 law. Michigan was the only state to have a court of appeals determine a local law that raised the MLSA was preempted by the state, and this was determined before the state Tobacco 21 bill was enacted into law. Eight states without clear preemption (Alabama, Georgia, Maryland, Michigan, Nebraska, Vermont, Virginia, and West Virginia) may be potentially preempted or include limitations based on the precedent set by state courts (see Table 3 for citations of case law). For example, Georgia recognized implied preemption in Gebrekidan v. City of Clarkston, 784 S.E.2d 373 (2016):

“In implied preemption, the intent of the General Assembly to preempt local regulation on the same subject as the general law is inferred from the comprehensive nature of the statutory scheme”.

However, in Alabama, Peak v. City of Tuscaloosa, 73 So.3d 5 (2011) cited past court decisions to prioritize public health as long as the laws were not perceived to be “clearly arbitrary and unreasonable” *Cudd v. City of Homewood*, 284 Ala. 268, 270, 224 So.2d 625, 627 (1969) and consistent with the general laws of the State, Ala. Const., Art. IV, § 89 (1901); Ala.Code § 11–45–1 (1975).

## 4. Discussion

This study provides an update to Berman’s 2014 assessment of state-level preemption of MLSA laws as well as a comprehensive assessment of state-level preemption using state statutes and case laws [4]. Before 2015, 19 of the 50 U.S. states preempted local authority and eight states expressly allowed localities to enact MLSA laws. Subsequently, 40 states have enacted a Tobacco 21 MLSA law, a count that does not include Mississippi’s Tobacco 21 law that only addressed the minimum purchase age for tobacco products. Among states that passed a Tobacco 21 MLSA law, seven introduced or expanded preemption and no states removed preemption as a part of their Tobacco 21 adoption. The Texas law provided a clear example of the overall expansion of state-level preemption and reduction of expressed local authority in the adoption of Tobacco 21. Furthermore, by including a review of case laws, we can better understand how courts may interpret state statutes, providing a clearer picture of the reality of preemptive language included in U.S. state MSLA laws.

Notably, California has been reported in previous studies as a state that preempted local MLSA [4,14]. By reviewing case laws, our findings differ from past research in that we found California’s preemption to only apply to the penal code, which does not apply to local ordinances, determined by state-level case law. Therefore, although the state statute may appear to include preemption, local ordinances can enact stricter MLSA laws than the state. For example, one court case defined the difference between local ordinances that regulate the legal age of sale through a tobacco retailer licensing structure as opposed to a local ordinance that creates a criminal penalty for the sale of tobacco products to be inconsistent with the penal code. In this case, a local ordinance banned the sale of tobacco products via vending machines, and the court reviewed whether the state legislature intended to occupy the field of tobacco retailer regulation. Ultimately, the court found there to be non-preemption clauses in multiple state tobacco laws (*O’Connell, supra,* 41 Cal.4th at p. 1068, 63 Cal.Rptr.3d 67, 162 P.3d 583). Although this case was not explicitly about a Tobacco 21 ordinance, it likely could be used as a precedent set within the state if a local MLSA was challenged on the grounds of preemption. Thus, this study is the first to recognize that California’s MLSA may not be preempted, as reported in previous studies [4,14].

State-level preemption of lower government authority creates barriers to the creation and evolution of tobacco control that often emerge from local laws [4,6]. While the enactment of state and federal Tobacco 21 laws are public health milestones, the long-term effects of expanded preemption of local authority in the seven states that included preemption in their Tobacco 21 law may be detrimental to the diffusion of comprehensive tobacco control. An analysis of smoke-free preemption laws revealed that once state preemption was established it took on average 11 years to repeal it, illustrating its long-term effects [6]. Furthermore, these findings may help to explain why Altria, the largest U.S. tobacco company, supported state Tobacco 21 bills that expanded preemption [14], efforts that mirror industry strategies employed in the 1990s to slow the expansion of effective tobacco control [11].

Despite these setbacks, encouraging progress occurred in Colorado where state legislators removed a purchase provision from their state law, and restricted localities from enacting minimum purchase age laws below 21 years. Purchase laws, which penalize the underage purchaser rather than the retailer, have been confused via social media with MLSA laws [15]. The removal of purchase laws is important from a normative perspective, as it encourages localities to adopt tobacco ‘sale’ laws rather than purchase, use, or possession (PUP) laws. PUP laws are problematic because penalties disproportionately affect vulnerable populations who are targeted by the tobacco industry’s marketing [16]. Furthermore, the language used within Colorado’s law may have created a minimum standard (floor preemption) rather than preemption of the law entirely. As the state prohibited localities from enacting purchase laws for those under 21 years of age, it created a minimum purchase age standard of 21 years; however, localities could technically raise the purchase age above 21 years. The use of punitive preemption has been seen in other areas of public health and may include threats for localities to enact strong public health laws; however, preempting purchase laws could also protect public health by ensuring that localities do not penalize youth who are a target market of the tobacco industry [17].

Policy language and definitions included within state MSLA laws are also important as they may determine how these laws are upheld in court. For example, Massachusetts’ state law included a time-limited deadline for action by the local authority, and 230 of 351 municipalities (that cover approximately 90% of the population) increased their MLSA by the deadline. Despite this, the state’s preemption could have further implications such as restricting jurisdictions from increasing the MLSA in the future. However, some jurisdictions have found alternative strategies to avoid such preemption by setting a maximum date of birth for sales of tobacco products instead of revising the MLSA. For example, Brookline, Massachusetts was the first U.S. city to adopt a tobacco-free generation law that prohibits tobacco sales to persons born after the year 2000 [18]. This tobacco endgame strategy exists in other countries and is now under consideration in California and Hawaii [19].

As explained earlier, some state courts have used ‘implied preemption’ to impede local tobacco control efforts despite the absence of clear preemption language in the state statutes [4]. Thus, until clear legal language expresses that local MLSA is not preempted by state law, local tobacco control policy efforts that strengthen MLSA enforcement in Alabama, Georgia, Maryland, Michigan, Nebraska, Vermont, Virginia, and West Virginia may not be successful. However, as noted in Table 1, Michigan may be one exception. Prior to the enactment of its state Tobacco 21 law, the Court of Appeals in Michigan found a local MLSA ordinance to be preempted by state law. Although not a ‘saving clause’, language in the state statute explains that following enactment of the Tobacco 21 law, the state law now supersedes state laws describing the rights and legal capacity of individuals 18–20 years old. Thus, this could be interpreted to include the Age of Majority Act. Michigan localities seeking to strengthen Tobacco 21 laws may have greater success following the enactment of their state law.

We found six states clearly expressed that state law does not preempt local authority, and in ten states, local laws had already been enacted. In these 16 states, advocates are encouraged to amend local Tobacco 21 laws in order to strengthen implementation and enforcement practices. Noted in previous studies, few local policies mandate enforcement components such as listing a minimum number of compliance checks per retailer, determining a minimum age for the decoy used during a compliance check, and providing education for retailers and the general public about the law [5]. While case law may not be advantageous for some states, court proceedings have upheld local authority in states such as Kansas (Dwagfys Manufacturing v. City of Topeka, 443 P.3d 1052 [2019]), providing foundational precedent for other municipalities seeking to pass local Tobacco 21 laws. Among states that have not raised their state MLSA to 21, some may be hesitant to amend it, with concern that the tobacco industry could use the opportunity to add preemption during the amendment process. Given the burden it takes to undo preemption, this concern is warranted; however, when preemption is vague, creative policy routes may allow localities to continue advancing tobacco control efforts. For example, although Massachusetts preempts local authority for MLSA laws, Brookline’s by-law does not apply to the MLSA [18].

Study strengths include the first comprehensive analysis of state MLSA laws since 2016 using a process that employed several levels of review (including a review of state case law). A limitation is the possibility that policy language was misinterpreted. Advocates, lawmakers, and policy experts may infer state laws or court decisions differently, and certainly, future court decisions may create a new precedent than that used in previous tobacco or alcohol-related law. Furthermore, the case law review in the current study is based on an initial analysis of current preemption and the outcome of a court proceeding would depend on the specific facts of the case, wording of the local ordinance, and ultimately the court review.

## 5. Conclusions

Despite advances in tobacco control policy efforts over the past decade, our findings indicate that preemption was introduced or expanded in seven new state tobacco MLSA laws, raising the total number of preempted states from 19 to 26. Prior evidence suggests that this included provision may hinder tobacco control efforts. Advocates seeking to update state MLSA laws should educate policymakers to avoid the inclusion of preemption. In the case where state laws already include preemption, advocates should seek to remove existing preemption that restricts localities from passing more stringent enforcement measures than the state. Removing preemption from state laws allows for the future development and diffusion of tobacco control efforts to address the ever-changing landscape of tobacco products. Additionally, state-level policymakers are encouraged to enact laws that clearly express no preemption of local authority. Findings from this study can help advocates determine if local authority would likely be allowed or upheld in court if enacted in their state. Moreover, this study highlights the expansion of preemption within state MLSA laws that has taken place over the past decade. In sum, policymakers should amend Tobacco 21 laws to ensure that they promote effective tobacco control efforts at the state and local levels.

## Figures and Tables

**Table 1:** US Tobacco Minimum Legal Sales Age Preemption Laws, As of 1 October 2022.

State	Raised MLSA to 21	Preemption Expressed	Expressly Not Preempted	Preemption Not Mentioned	Citations(s)
AL	✓			✕	
AK				✕	
AZ				✕	
AR	✓	✕ *		- - -	§ 26-57-259 (a)(2)(A)
CA	✓	✕ ***			Pen.Code, § 308
CO	✓		✕		§ 18-13-121; 25-14-301
CT	✓			✕	
DE	✓	✕			11 Del.C. § 1127
FL	✓	✕ *		- - -	§§ 569.0025; 569.315
GA	✓			✕	
HI	✓	✕ *	- - -		HI Rev. Stat. § 328J-115
ID	✓	✕ *	- - -		§ 39-5713
IL	✓			✕	
IN	✓	✕			§§ 16-41-39-1; 16-41-39-2
IA	✓	✕			I.C.A. § 453A.56
KS				✕	
KY	✓	✕			KRS § 438.300
LA	✓	✕			LA Rev. Stat. § 91.8.B
ME	✓		X		ME ST T. 22 § 1555-B
MD	✓			X	
MA	✓	✕ *		- - -	2018 Mass. Legis. Serv. Ch. 157 (H.B. 4486)
MI	✓			✕ **	
MN	✓		✕		MN ST § 609.685
MS ^†^		✕			Miss. Code Ann. § 97-32-2
MO			✕		Mo. St. § 407.923
MT		✕			Mt. St. Code § 16-11-311
NE	✓			✕	
NV	✓	✕			Nev. Rev. St. § 202.249
NH	✓		✕		N.H. Rev. St. § 126-K:14
NJ	✓			✕	
NM	✓	✕			N.M. St. Ann. § 61-37-24
NY	✓			✕	
NC		✕			N.C. St. Ann. § 14-313(e)
ND	✓		✕		N.D. St. Ann. § 12.1-31-03
OH	✓			✕	
OK	✓	✕			Okla. St. Ann. § 1-229.13
OR	✓			✕	
PA	✓	✕			PA. St. tit. 72 § 232-A
RI	✓			✕	
SC		✕			S.C. Code Ann. § 16-17-504
SD	✓	✕			S.D. Code § 34-46-6/
TN	✓	✕			Tenn. Code Ann. § 39-17-1551
TX	✓	✕ *	- - -		Health & Safety Code § 161.089
UT	✓	✕ *		- - -	Utah St. Ann. § 76-10-116
VT	✓			✕	
VA	✓			✕	
WA	✓	✕			Wa. Rev. Code § 70.155.130
WV				✕	
WI		✕			WI. St. Ann. § 134.66(5)
WY	✓	✕			Wy. St. Ann. § 14-3-308
Total	40	26	6	18	

Note. * Introduced new or expanded language on preemption of tobacco control. ** Not expressly preempted in statute, but courts have interpreted the Age of Majority Act to invalidate local MLSA Tobacco 21 laws. *** Preemption included in penal code but does not apply to local ordinances based on case law. - - - Statute changed from this as a part of T21 legislation adoption. † Law raised purchase age for tobacco products and the MLSA for alternative nicotine products, but not the MLSA of all tobacco products.

**Table 2:** Example language from state MLSA tobacco laws in the US.

Preemption of Local Authority
A political subdivision may not adopt or enforce an ordinance or requirement relating to the lawful age to sell, distribute, or use cigarettes, e-cigarettes, or tobacco products that is more stringent than a requirement prescribed by this subchapter—Texas; Tex. Health & Safety Code Ann. §§ 161.089 (2019)
**Expressed that localities could enact stricter laws than the state**
Nothing in subdivisions 1 to 2a shall supersede or preclude the continuation or adoption of any local ordinance which provides for more stringent regulation of the subject matter in subdivisions 1 to 2a—Minnesota; MINN. STAT. § 609.685 (4) (2020)
**Transfer of purchase law to sales law**
Nothing in this section prohibits a statutory or home rule municipality, county, or city and county from enacting an ordinance or resolution that prohibits the SALE of any cigarettes, tobacco products, or nicotine products to person under TWENTY-ONE years of age or imposes requirements more stringent than provided in this section. COLO. Rev. STAT §§ 18-13-121(3) (2020)
**Prohibition of purchase laws for those under 21 years of age**
A Statutory or home rule municipality, county, or city and county shall not enact an ordinance or resolution that establishes a minimum age to purchase cigarettes, tobacco products, or nicotine products that is under twenty-one years of age—Colorado; COLO. Rev. STAT. §§ 18-13-121(3) (2020)

**Table 3:** State Case Law Preemption Threats.

State	Citations from Courts Decisions about Preemption Analysis
Alabama	Peak v. City of Tuscaloosa, 73 So.3d 5 (2011)
Georgia	Gebrekidan v. City of Clarkston, 784 S.E.2d 373 (2016)
Maryland	Altadis v. Prince George Cty MD, 65 A.3d 118 (2013)
Michigan *	Age of Majority Act (Mich. § 722.52)
Nebraska	Malone v. City of Omaha, 883 N.W.2d 516 (2016)
Vermont	North Country Sportsman’s Club v. Town of Williston, 170 A.3d 639 (2017)
Virginia	Va. Code Ann. § 1-248
West Virginia	American Tower Corp. v. Common Council City of Beckley, 557 S.E.2d 752 (2001)

* Court of Appeals found that a local ordinance raising the MLSA was preempted by state law; However, language included within the Tobacco 21 law may supersede the Age of Majority Act.

## Data Availability

Data will be made available upon request.

## References

[1] Bonnie R.J., Stratton K., Kwan L.Y., Institute of Medicine (2015). Public Health Implications of Raising the Minimum Age of Legal Access to Tobacco Products.

[2] Centers for Disease Control and Prevention STATE System Tobacco MLSA Fact Sheet. https://www.cdc.gov/statesystem/factsheets/mlsa/Minimum-Legal-Sales-Age.html?CDC_AA_refVal=https%3A%2F%2Fwww.cdc.gov%2Fstatesystem%2Ffactsheets%2Ft21%2FTobacco21.html.

[3] Preventing Tobacco Addiction Foundation State By State Tobacco Laws, Taxes, and Statistics. https://tobacco21.org/state-by-state/.

[4] Berman M.L. (2016). Raising the tobacco sales age to 21: Surveying the legal landscape. Public Health Rep..

[5] Dobbs P.D., Chadwick G., Dunlap C.M., White K.A., Cheney M.K. (2021). Tobacco 21 policies in the U.S.: The importance of local control with federal policy. Am. J. Prev. Med..

[6] Crosbie E., Schmidt L.A. (2020). Preemption in tobacco control: A framework for other areas of public health. Am. J. Public Health.

[7] Carr D., Adler S., Winig B.D., Montez J.K. (2020). Equity first: Conceptualizing a normative framework to assess the role of preemption in public health. Milbank Q..

[8] Weber L. (2007). Policing the Virtual Border: Punitive preemption in Australian offshore miggration control. Soc. Justice.

[9] Crosbie E., Pomeranz J.L., Wright K.E., Hoeper S., Schmidt L. (2021). State preemption: An emerging threat to local sugar-sweetened beverage taxation. Am. J. Public Health.

[10] Crosbie E., Hatefi A., Schmidt L. (2019). Emerging threats of global preemption to nutrition labelling. Health Policy Plan..

[11] U.S. Department of Health and Human Services (2006). The Health Consequences of Involuntary Exposure to Tobacco Smoke: A Report of the Surgeon General.

[12] ChangeLab Solutions Assessing & Addressing Preemption. https://www.changelabsolutions.org/product/assessing-addressing-preemption.

[13] Sykes J.B., Vanatko N. (2019). Federal Preemption: A Legal Primer.

[14] Dobbs P.D., Chadwick G., Ungar K.W., Dunlap C.M., White K.A., Kelly M.C.T., Cheney M.K. (2020). Development of a tobacco 21 policy assessment tool and state-level analysis in the USA, 2015–2019. Tob. Control.

[15] Dobbs P.D., Schisler E., Colditz J.B., Primack B.A. (2022). Miscommunication about the US federal Tobacco 21 law: A content analysis of Twitter discussions. Tob. Control.

[16] Tobacco Control Enforcement for Racial Equity: Decriminalizing Commercial Tobacco Addressing Systemic Racism in the Enforcement of Commercial Tobacco Control. https://sph.cuny.edu/wp-content/uploads/2020/10/Tobacco-Control-Enforcement-for-Racial-Equity_FINAL_20201007.pdf.

[17] U.S. Department of Health and Human Services (2012). Preventing Tobacco Use among Youth and Young Adults: A Report of the Surgeon General.

[18] Ducharme J. (2021). How One Massachusetts Town Could Shape the Future of Tobacco.

[19] Sheeler A. CA Bill Would Bar Tobacco Sales to Anyone Born in 2007 or Later. The Sacramento Bee. https://www.sacbee.com/news/politics-government/capitol-alert/article272538573.html.

